# Cutaneous fistula on the cheek associated with oculo-auriculo-vertebral spectrum

**DOI:** 10.1093/jscr/rjac187

**Published:** 2022-05-24

**Authors:** Soh Nishimoto, Kenichiro Kawai, Toshihiro Fujiwara, Hisako Ishise, Masao Kakibuchi

**Affiliations:** Department of Plastic Surgery, Hyogo College of Medicine, Nishinomiya, Hyogo, Japan; Department of Plastic Surgery, Hyogo College of Medicine, Nishinomiya, Hyogo, Japan; Department of Plastic Surgery, Hyogo College of Medicine, Nishinomiya, Hyogo, Japan; Department of Plastic Surgery, Hyogo College of Medicine, Nishinomiya, Hyogo, Japan; Department of Plastic Surgery, Hyogo College of Medicine, Nishinomiya, Hyogo, Japan

## Abstract

A Japanese boy, presented with epibulbar dermoid and ipsilateral preauricular appendages, had a pit on his cheek of the same side. An atrial septal defect and vertebral fusions were also identified. He was diagnosed with a mild type of oculo-auriculo-vertebral spectrum (OAVS). At the age of 18 months, his cheek was swollen with a slight fever. An infected cyst and cutaneous fistula enveloped by the risorius muscle were extracted. It was assumed to be a remnant of the fissure between the maxillary and mandibular prominences. This was the first case of cutaneous fistula confirmed histologically with OAVS, although there seem to be more cases. The possibility of the mechanism of smiling cheek dimple is also discussed.

## INTRODUCTION

Oculo-auriculo-vertebral spectrum (OAVS) is a group of conditions that involve eye, ear and cheek, with a wide variety of severity [1]. Cranio-facial anomalies with OAVS are believed to be caused by the abnormal developments of the first and second pharyngeal arches. A case of cutaneous fistula on Tessier’s No. 7 cleft line with a mild type of OAVS was encountered.

## CASE REPORT

**Figure 1:**
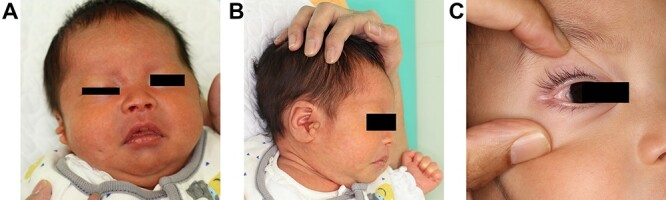
(**A**) A 2-month-old baby presented a small pit outside of his right oral commissure. The difference between the left and right cheeks was not remarkable. (**B**) There presented preauricular appendages on his right side. (**C**) There presented an epibulbar dermoid inside of his right external eye angle.

A Japanese male baby presented with a pit on his right cheek (Fig. 1). No discharge from the pit was observed. Preauricular appendages and an epibulbar dermoid on the eyeball were observed on the ipsilateral side. The obvious difference between right and left cheeks or mandibles was not presented. An atrial septal defect was pointed out by ultrasonography, which eventually closed. X-ray observation pointed out fusions between second and third, fourth and fifth cervical spines. These observations categorized him as a mild type of OAVS. Although his parents had no specific symptoms, his eldest sister had auditory disturbance on the right side. The second elder sister had right microtia. The parents did not want genetic testing.

**Figure 2:**
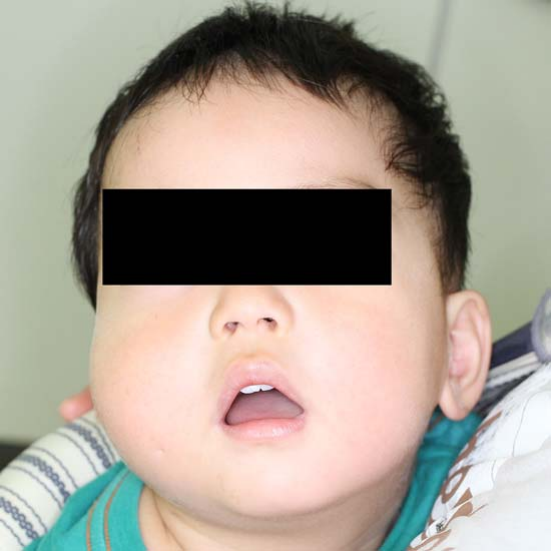
At the age of one and a half, his right cheek was swollen with a slight fever. No discharge from the pit was observed.

**Figure 3:**
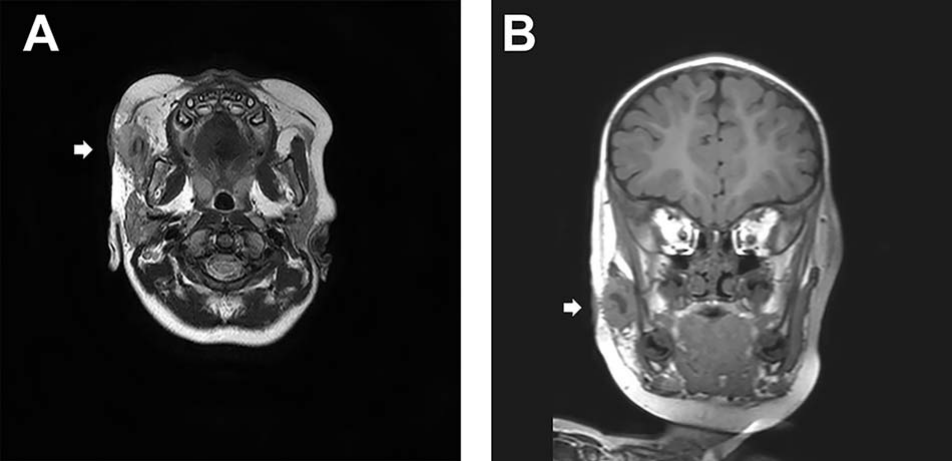
(**A**) A transverse sT2W mDixon MRI image. A cystic lesion beneath the superficial musculo-aponeurotic system is identified. (**B**) A coronal section of MRI.

At the age of one and a half, his right cheek was swollen with a slight fever (Fig. 2). magnetic resonance imaging (MRI) presented a cystic lesion beneath the superficial musculo-aponeurotic system (Fig. 3). Although antibiotic was administered, it ruptured to the skin during the preparation period for the operation. *Staphylococcus aureus* was detected in the pus.

**Figure 4:**
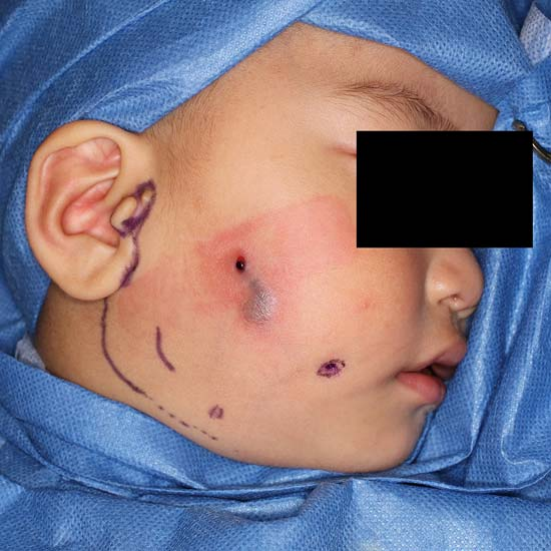
Christal violet ink was injected from the cheek pit. The ink came out from the perforated hole. An elongated retro-mandibular incision was designed.

**Figure 5:**
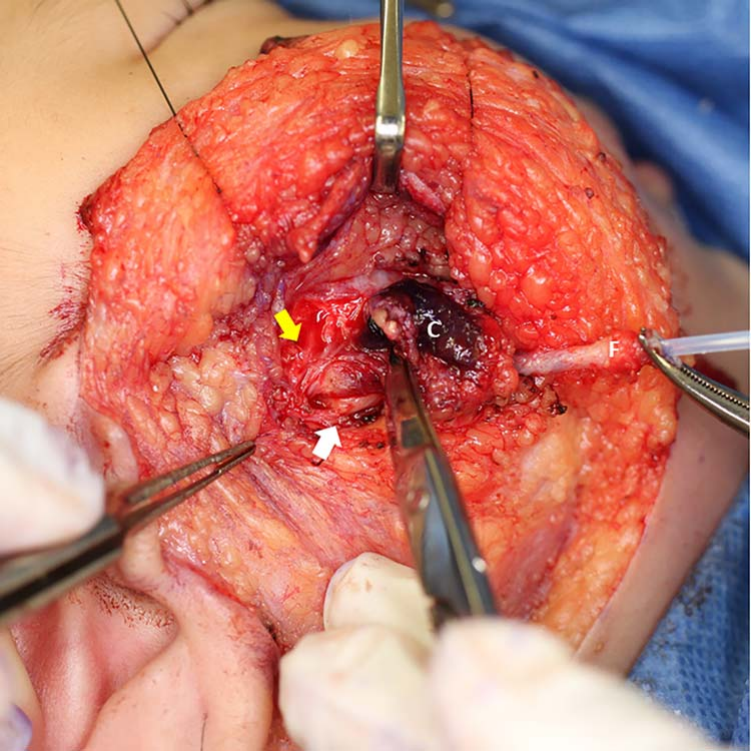
The yellow arrow points one of the buccal branches of facial nerve. The white arrow points Steensen’s duct. C: cyst, F: fistula, canulated with a plastic outer tube of an intravenous catheter.

**Figure 6:**
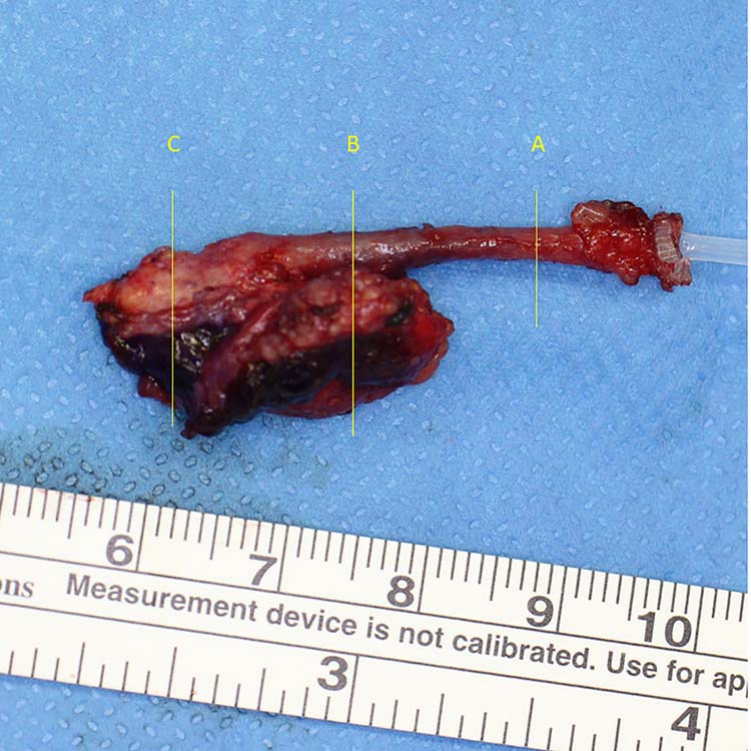
Extracted fistula and cyst. A, B, C indicate cross-sections for histology.

**Figure 7:**
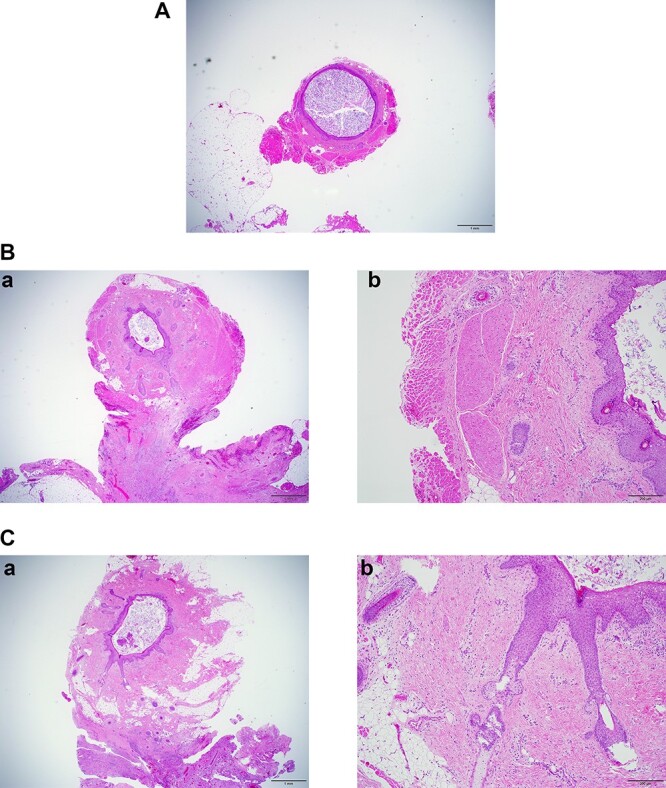
(**A**) Section at A in Fig. 6. ×2 objective lens. The fistula wall consists of squamous cells. The lumen is filled with cornified material. (**B-a**) Section at B in Fig. 6. ×2 objective lens. Many hair follicles can be seen. Adjacent to the fistula, chronic inflammation is observed. (**B-b**) Section at B in Fig. 6. ×10 objective lens. The fistula is surrounded by skeletal muscle. (**C-a**) Section at C in Fig. 6. ×2 objective lens. At this section level, the fistula is not accompanied by muscles. (**C-b**) Section at C in Fig. 6. ×10 objective lens. Long hair follicles and glands can be observed.

## OPERATION

Under general anesthesia, the ablative operation was performed. Christal violet ink was injected into the cheek pit. The dye dyed subcutaneous induration and came out from the perforated site (Fig. 4). Incision line was put on the retro-mandibular line as parotidectomy, elongated cephalically to excise the preauricular appendage. The skin flap was raised superficially to the platysma. The skin on the infected cyst was preserved as much as possible. The duct leading from the pit was dissected. It descended from the subcutaneous fat layer to modiolus, run laterally, enveloped by risorius muscle. The corner of his mouth was yanked as the fistula was pulled (Supplementary material). The infected cyst lay on the masseter muscle. Stensen’s duct was observed beneath the cyst. A zygomatic branch of the facial nerve was identified with a nerve stimulator and preserved (Fig. 5). The duct ended in a blind end (Fig. 6).

## HISTOLOGIC FINDINGS

The fistula wall was lined with keratinized squamous epithelium with appendages as hair follicles and glands. The lumen was filled with laminar cornified material. The central part of the fistula was surrounded by skeletal muscle. Adjacent to the fistula, chronic abscess with foreign body reaction for ruptured fistula was observed (Fig. 7).

## DISCUSSION

In 1843, Thomson mentioned that the malformation of the external and middle ear is related to ‘two anterior branchial arches and the fissure between them’ [2]. After that, many articles discussed the etiological relationship between the first and second branchial (pharyngeal) arches and facial malformations [3]. The fissure between them is called the first pharyngeal cleft, and the abnormal fuse will leave cysts or fistula [4]. The external ear develops around the lateral end of the cleft [5]. The fissure between maxillary and mandibular prominences comprises the first pharyngeal arch, corresponding to Tessier’s No. 7 cleft line [6]. Goldenhar initially described a complication of ear and eye malformation with mandibulo-facial dysostosis [7]. Oculo-auriculo-vertebral dysplasia was a term suggested by Gorlin [8] adding vertebral abnormality as a sign. It may be called Goldenhar–Gorlin’s syndrome. Phenotype and severity vary in this spectrum [1].

In normal ontogeny, stomodeum can be seen around 23 days (4 weeks) of gestation [9]. Both maxillary and mandibular prominences are also identified at this stage. The fissure between them becomes wide initially during 5-week period. It fuses from the lateral end as the maxillary prominences immigrate medially and merge with the medial and lateral nasal prominences. Abnormal fusion of this fissure leaves transverse facial cleft or macrostomia. It seems likely that the same situation may leave a cutaneous fistula as this presented case. Though, within our literature search, there has been no other case.

Some reports about congenital cheek fistula with accessory parotid system accompanied by ipsilateral preauricular appendage, suggested as a variant of the oculo-auriculo-vertebral spectrum [10, 11]. The appearance and location of these fistulae resemble our case. Though, in our case, histologically, the fistula wall was composed of keratinized squamous epithelium with appendages, which was different from the parotid system. The parotid gland system develops, in the 6 weeks, from a thickening of the oral ectoderm at the most lateral point of the buccal groove, cranial to the angle of the mouth [12]. There may be a relationship of embryological etiology between those cases and the present case.

Pessa *et al*. found in some cadavers that the inferior slip of the double zygomatic muscle was inserted into the dermis of cheek skin [13]. They attribute the finding to the mechanism for the occurrence of cheek dimple. In our case, the surgical and histological findings indicated that the fistula ran into the modiolus and was enveloped by the risorius muscle. The vestige of the fissure between the maxillary and mandibular prominences may act as a tethering ligament or fascia between modiolus and skin, which also might play a role in cheek dimple appearing.

## CONFLICT OF INTEREST STATEMENT

None declared.

## FUNDING

None.

## Supplementary Material

asup1_rjac187
